# Supplementary figures and images for: Quantification of T-cell dynamics during latent cytomegalovirus infection in humans
Source: PLoS Pathog. 2021 Dec 16;17(12):e1010152. doi: 10.1371/journal.ppat.1010152 (PMC8717968; doi:10.1371/journal.ppat.1010152)

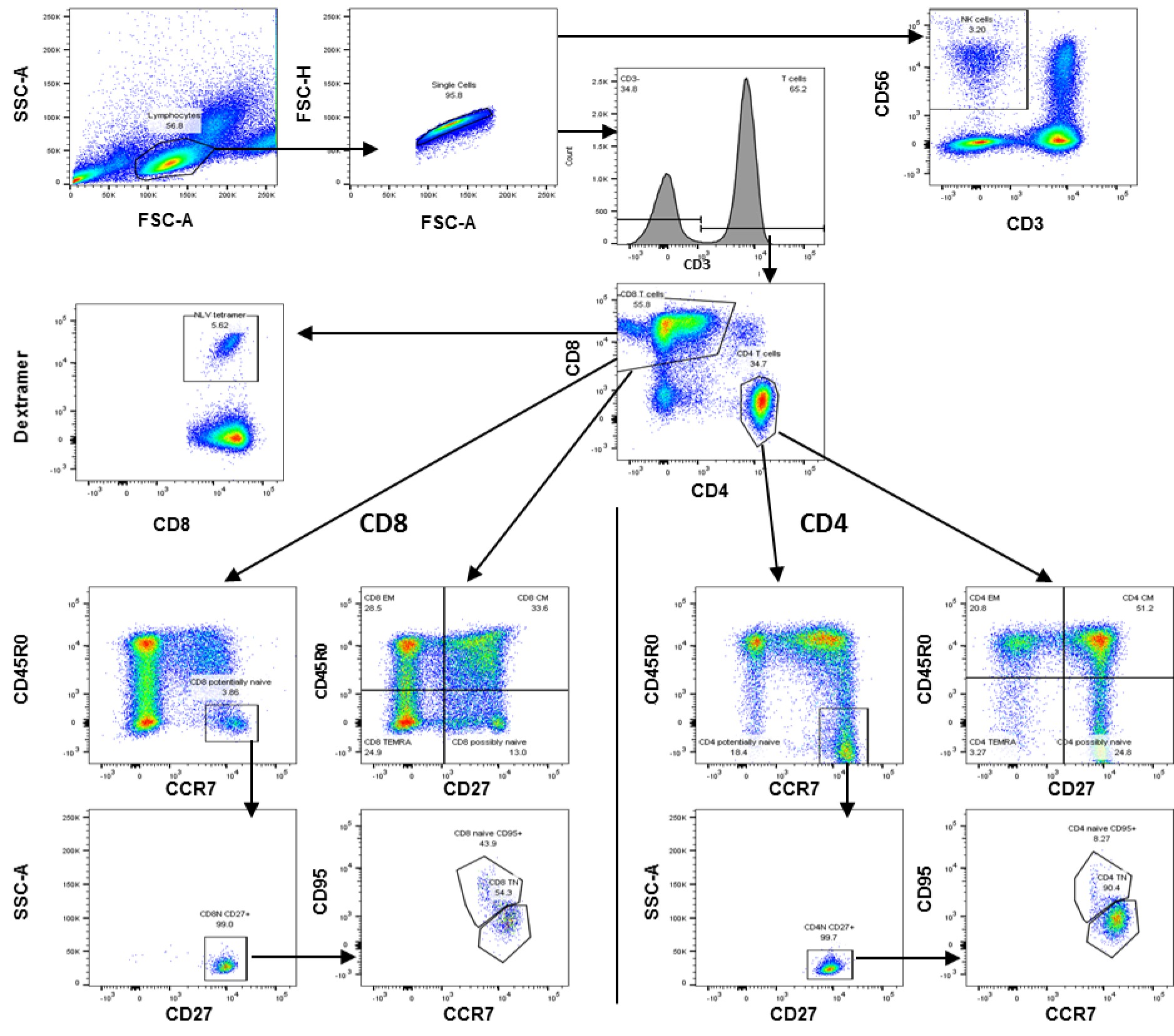

Supplement: S1 Fig — Peripheral blood mononuclear cells (PBMCs) were isolated using Ficoll-Paque PLUS density centrifugation and stained for CD3, CD56, CD4, CD8, CD45RO, CCR7, CD27, and CD95 (see Materials and methods for antibody specificities). Live lymphocytes were gated based on SSC-A/FSC-A and FSC-H/FSC-A plots. T-cells were subsequently defined as CD3+CD56-. T-cell subpopulations were defined as follows: truly naive, TTN (CCR7+CD45RO-CD27+CD95+), central memory, TCM (CD45RO+CD27+), effector memory, TEM (CD27-CD45RO+) and effector memory re-expressing RA, TEMRA (CD27-CD45RO-). (TIF) [file ppat.1010152.s001.tif]

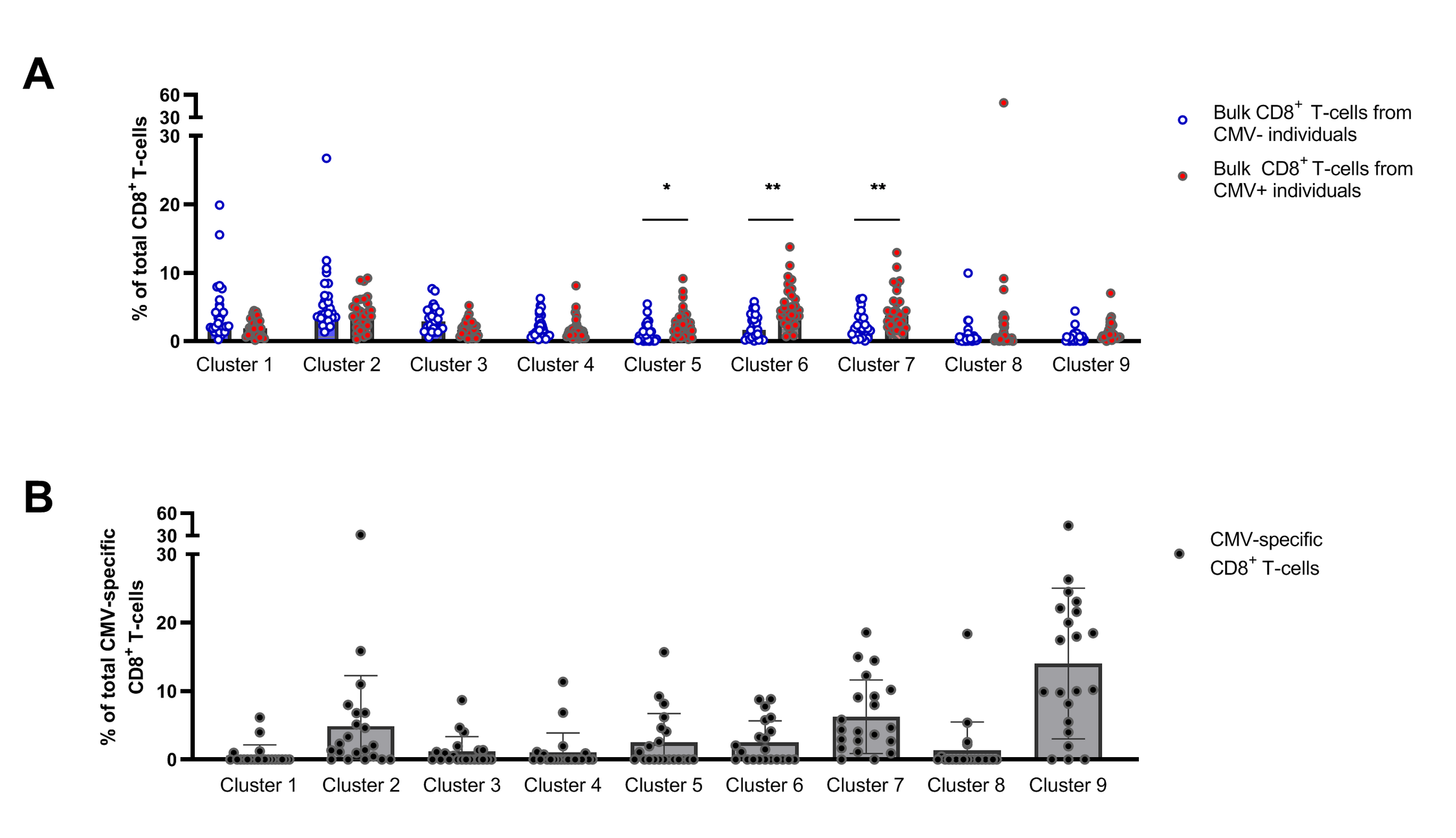

Supplement: S2 Fig — CD8+ T-cells of CMV- and CMV+ individuals were clustered by t-SNE analysis based on the expression of CD57, KLRG-1, CD127, CD27, CD45RO, CCR7, and CD95 (Fig 1E). Nine clusters were identified within the t-SNE plot based on cell density. (A) The percentage of CD8+ T-cells out of total CD8+ T-cells that fall into each cluster is shown for CMV- and CMV+ individuals. (B) The percentage of CMV-specific CD8+ T-cells out of total CMV-specific CD8+ T-cells that fall into each cluster is shown. We only included data points when more than 20 cells could be analyzed. Differences between CMV- and CMV+ individuals were tested by Kruskal-Wallis test. Stars indicate significant differences as follows: * P-value <0.05, ** P-value <0.01, *** P-value <0.001, *** P-value <0.0001. (TIF) [file ppat.1010152.s002.tif]

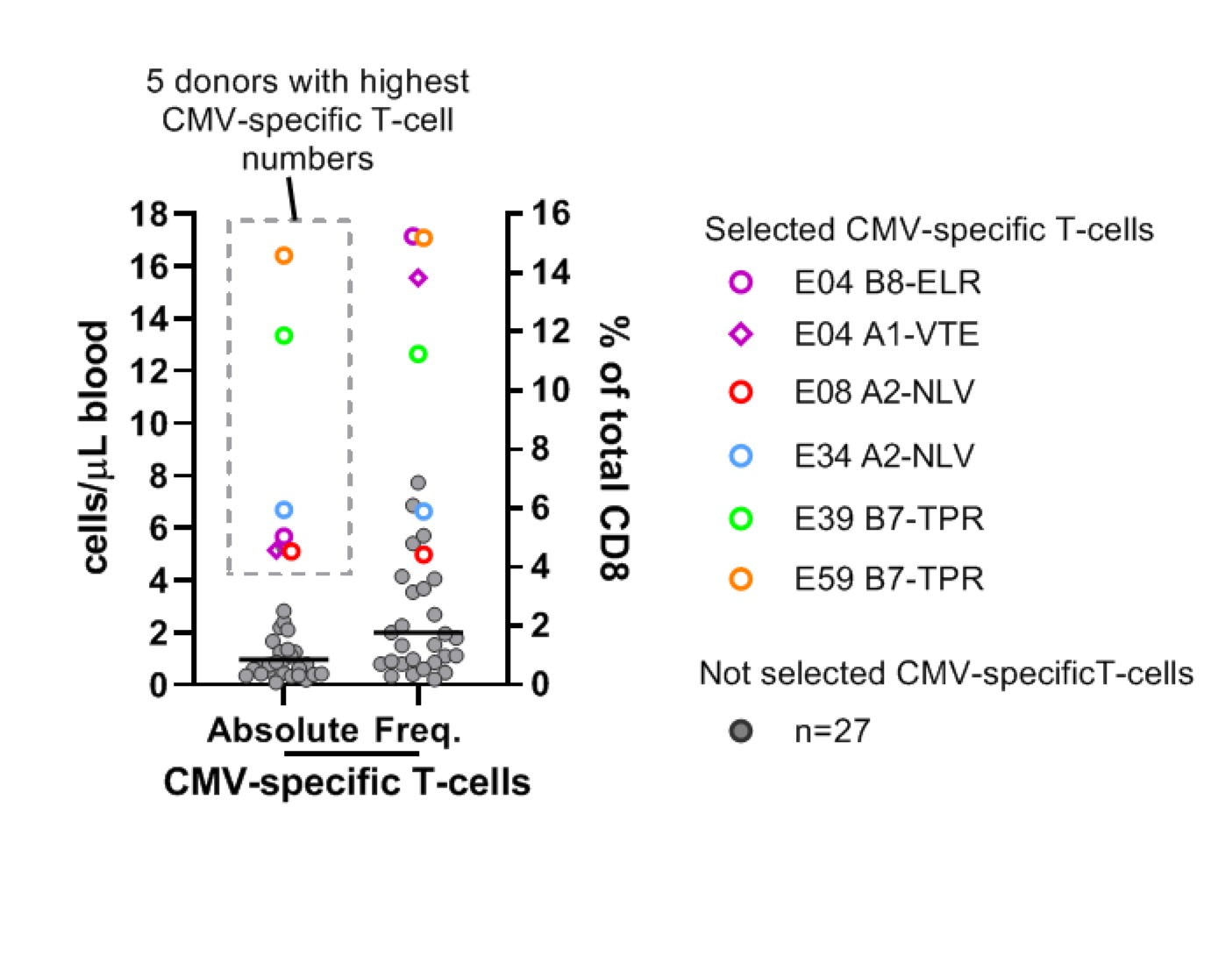

Supplement: S3 Fig — Absolute numbers (in cells/μL) and frequency (in % of total CD8+ T-cells) of CMV-specific CD8+ T-cells are shown for all 32 CMV+ individuals. For the heavy water labelling study, five CMV+ participants were selected based on highest absolute CMV-specific T-cell numbers (shown with individual symbol). CMV+ participants that were not selected are shown in grey. (TIF) [file ppat.1010152.s003.tif]

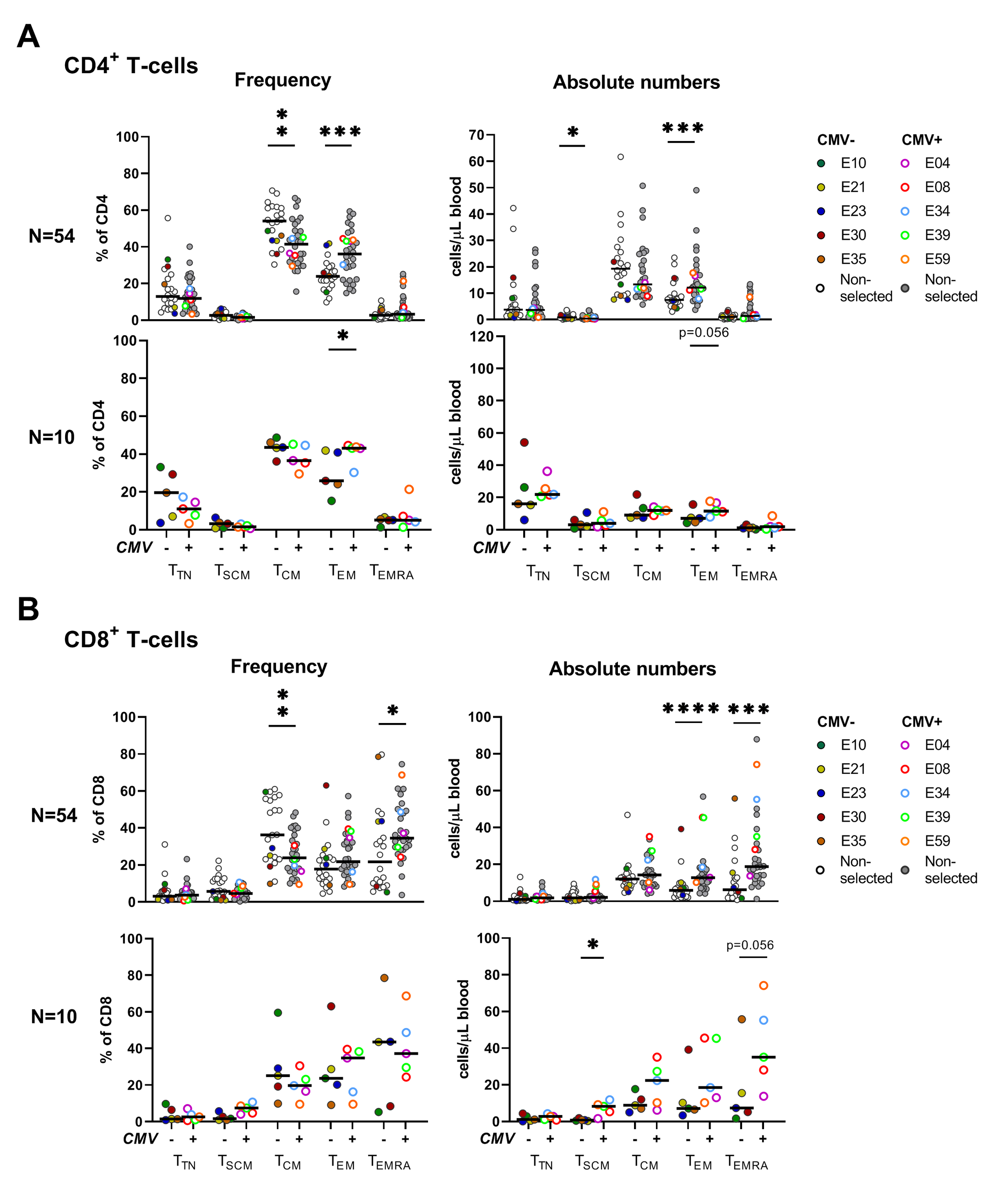

Supplement: S4 Fig — (A) and (B) The frequency (left panels) and absolute numbers (right panels) of different CD4+ (A) or CD8+ (B) T-cell populations. In the top panels of both (A) and (B) all participants are shown, and in the lower panels only those selected for the heavy water labelling study, highlighted with a unique color. Differences between CMV- and CMV+ individuals were tested by Mann-Whitney U test. Stars indicate significant differences as follows: * P-value <0.05, ** P-value <0.01, *** P-value <0.001, *** P-value <0.0001. (TIF) [file ppat.1010152.s004.tif]

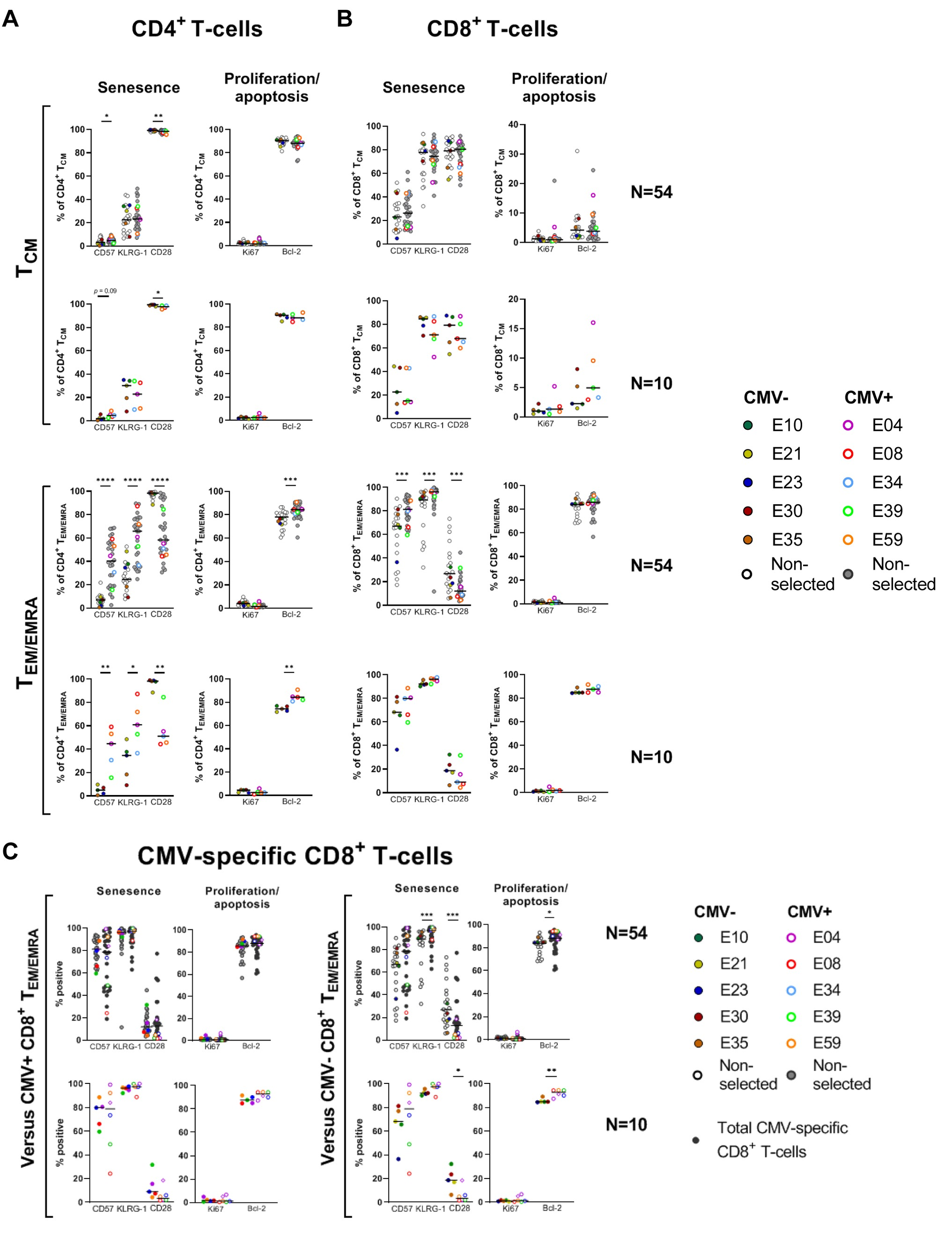

Supplement: S5 Fig — (A) and (B) The percentage of TCM and TEM/EMRA positive for CD57, KLRG-1, CD28, Ki-67, and Bcl-2 are shown for CD4+ (A) or CD8+ (B) T-cell populations. In the top panels of both (A) and (B) all participants are shown, and in the lower panels only those selected for the heavy water labelling study, highlighted with a unique color. (C) The expression of CD57, KLRG-1, CD28, Ki-67, and Bcl-2 on CMV-specific CD8+ T-cells is compared to the expression on bulk TEM/EMRA CD8+ T-cells from CMV+ individuals. Differences between CMV- and CMV+ individuals were tested by Mann-Whitney U test. Stars indicate significant differences as follows: * P-value <0.05, ** P-value <0.01, *** P-value <0.001, *** P-value <0.0001. (TIF) [file ppat.1010152.s005.tif]

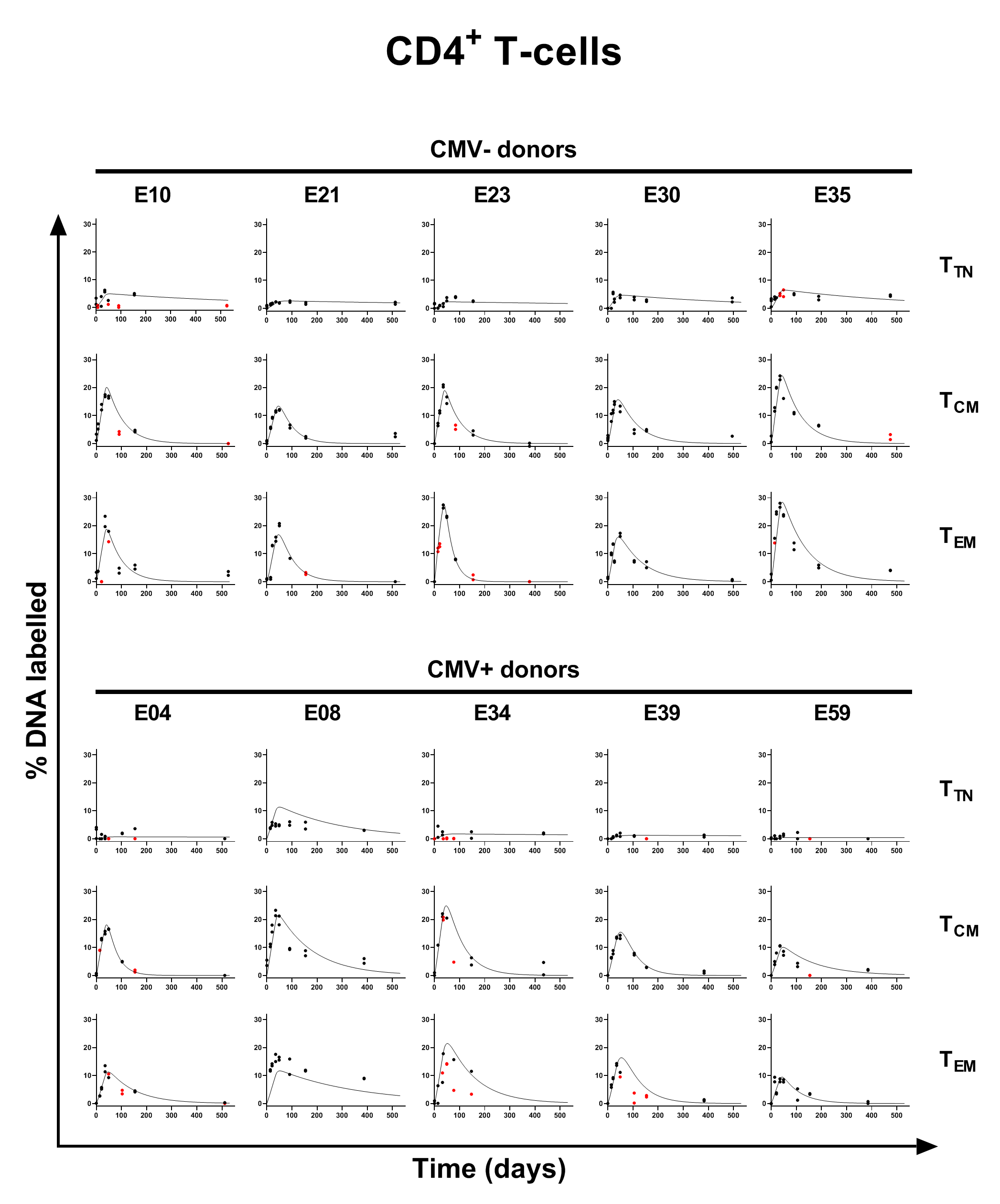

Supplement: S6 Fig — Best fits of the kinetic heterogeneity model to the enrichment in true naïve (TTN), central memory (TCM), and effector memory (TEM/EMRA) CD4+ T-cells. Label enrichment in the DNA was scaled between 0 and 100% by normalizing for the maximum enrichment in granulocytes (see Materials and methods). The measurements that were out of range of the standards are indicated in red closed symbols, those in range are indicated in black closed symbols. E35, E30, E23, E21, and E10 are CMV- individuals, E59, E39, E34, E08, and E04 are CMV+ individuals. E30 only received 2H2O for 3.5 weeks. (TIF) [file ppat.1010152.s006.tif]

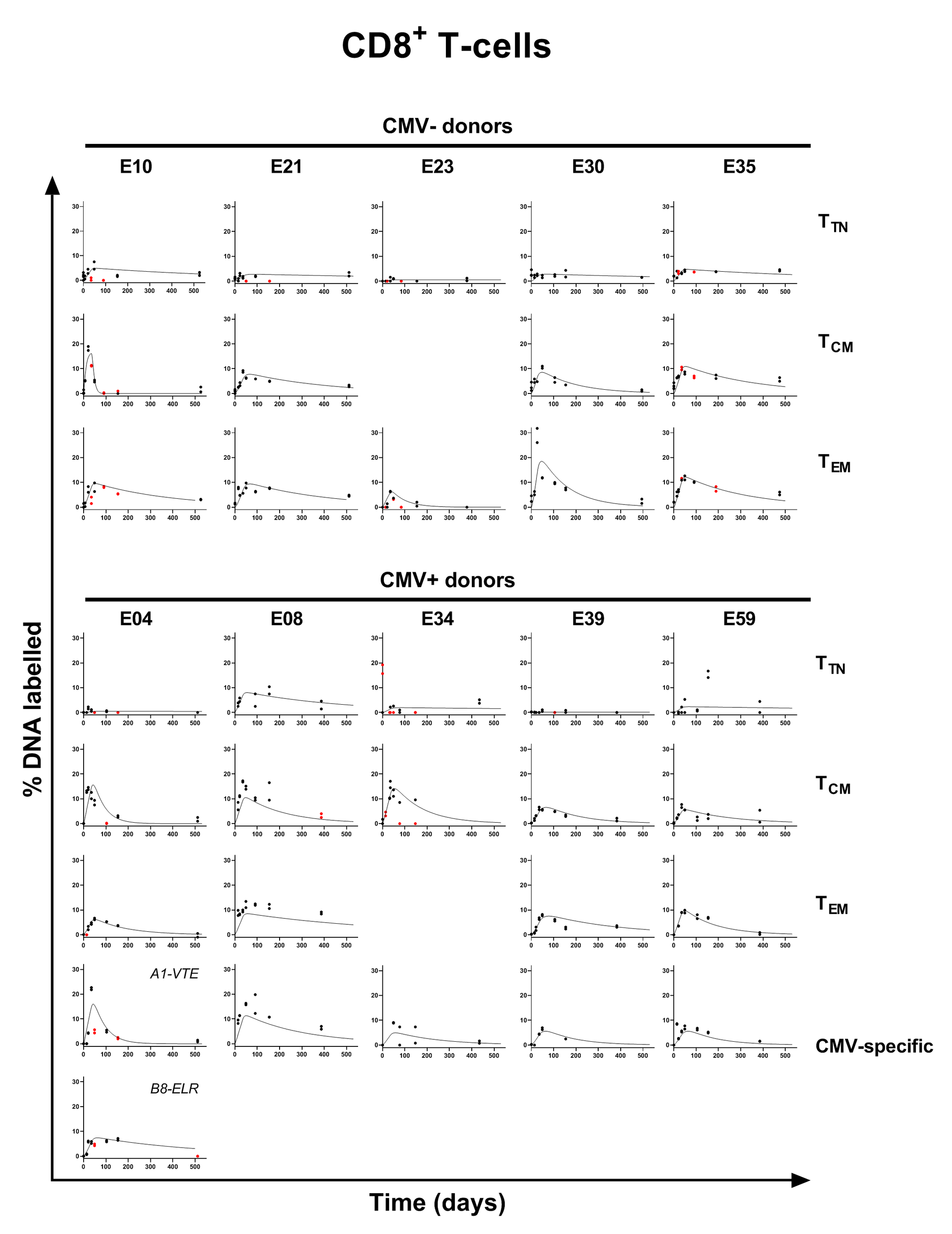

Supplement: S7 Fig — Best fits of the kinetic heterogeneity model to the enrichment in true naïve (TTN), central memory (TCM), effector memory (TEM/EMRA), and CMV-specific CD8+ T-cells. Label enrichment in the DNA was scaled between 0 and 100% by normalizing for the maximum enrichment in granulocytes (see Materials and methods). The measurements that were out of range of the standards are indicated in red closed symbols, those in range are indicated in black closed symbols. E35, E30, E23, E21, and E10 are CMV- individuals, E59, E39, E34, E08, and E04 are CMV+ individuals. E30 only received 2H2O for 3.5 weeks. (TIF) [file ppat.1010152.s007.tif]

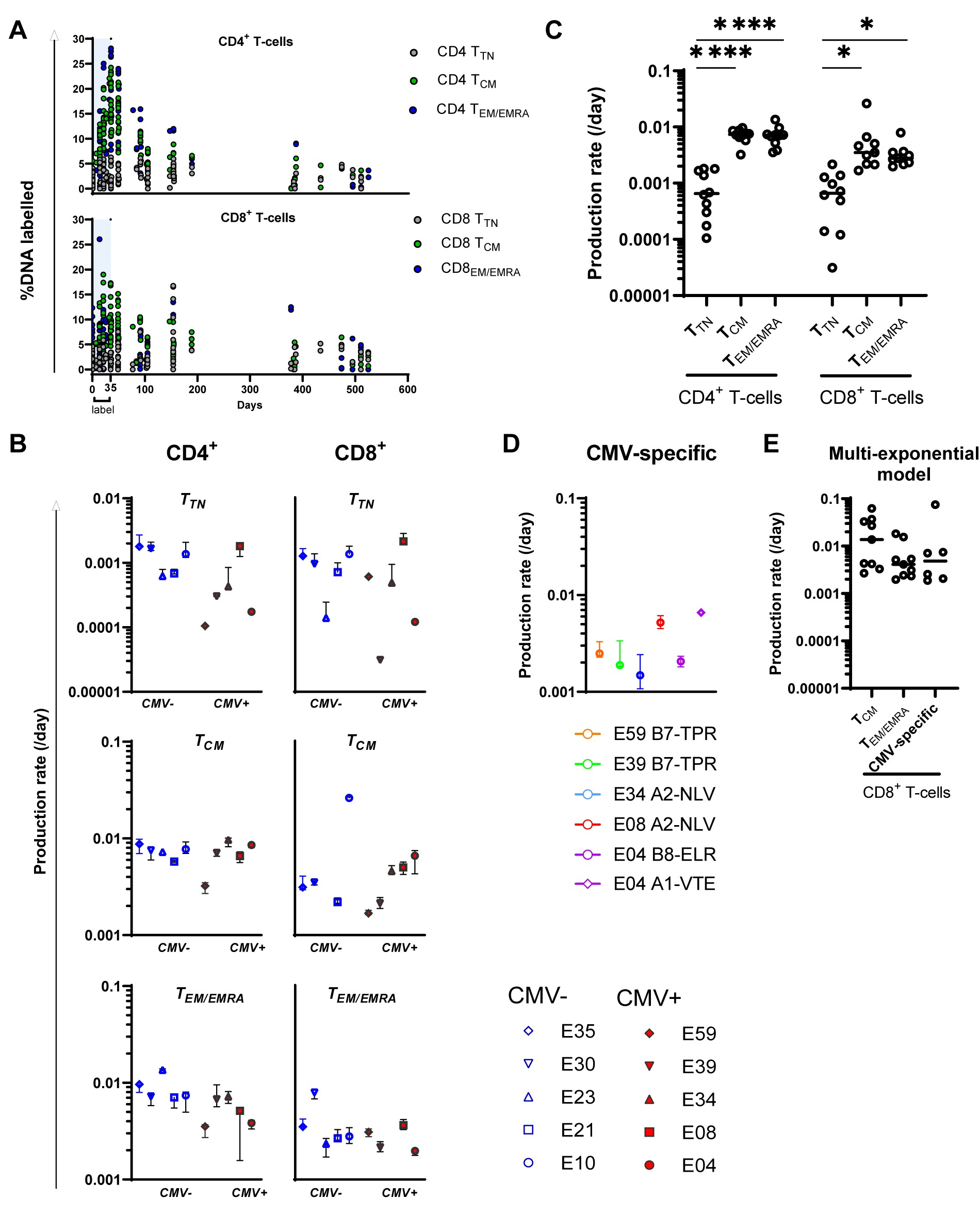

Supplement: S8 Fig — (A) Combined deuterium labelling enrichment (%DNA labelled) of different T-cell subpopulations for all individuals, both CMV+ and CMV-. Label enrichment was scaled between 0 and 100% by normalizing for the estimated maximum enrichment of granulocytes (see Materials and methods). (B) Summary of estimated production rates of TTN, TCM, and TEM/EMRA CD4+ and CD8+ T-cells in all individuals. (C) Summary of estimated production rates with confidence intervals of TTN, TCM, and TEM/EMRA CD4+ and CD8+ T-cells in CMV- (blue symbols) and CMV+ (red symbols) individuals. Data from each individual are represented by unique symbols. (D) Summary of estimated production rates with confidence intervals of CMV-specific CD8+ T-cells. All estimates in (B), (C), and (D) were obtained by fitting a single-exponential model to the data sets per individual (see Materials and methods and S6 Fig). E30 only received 2H2O for 3.5 weeks. (E) Summary of estimated production rates of TCM, TEM/EMRA and CMV-specific CD8+ T-cells by fitting a multi-exponential model to the data sets per individual. Differences between groups were assessed by Kruskal-Wallis test. Stars indicate significant differences as follows: * P-value <0.05, ** P-value <0.01, *** P-value <0.001, *** P-value <0.0001. (TIF) [file ppat.1010152.s008.tif]

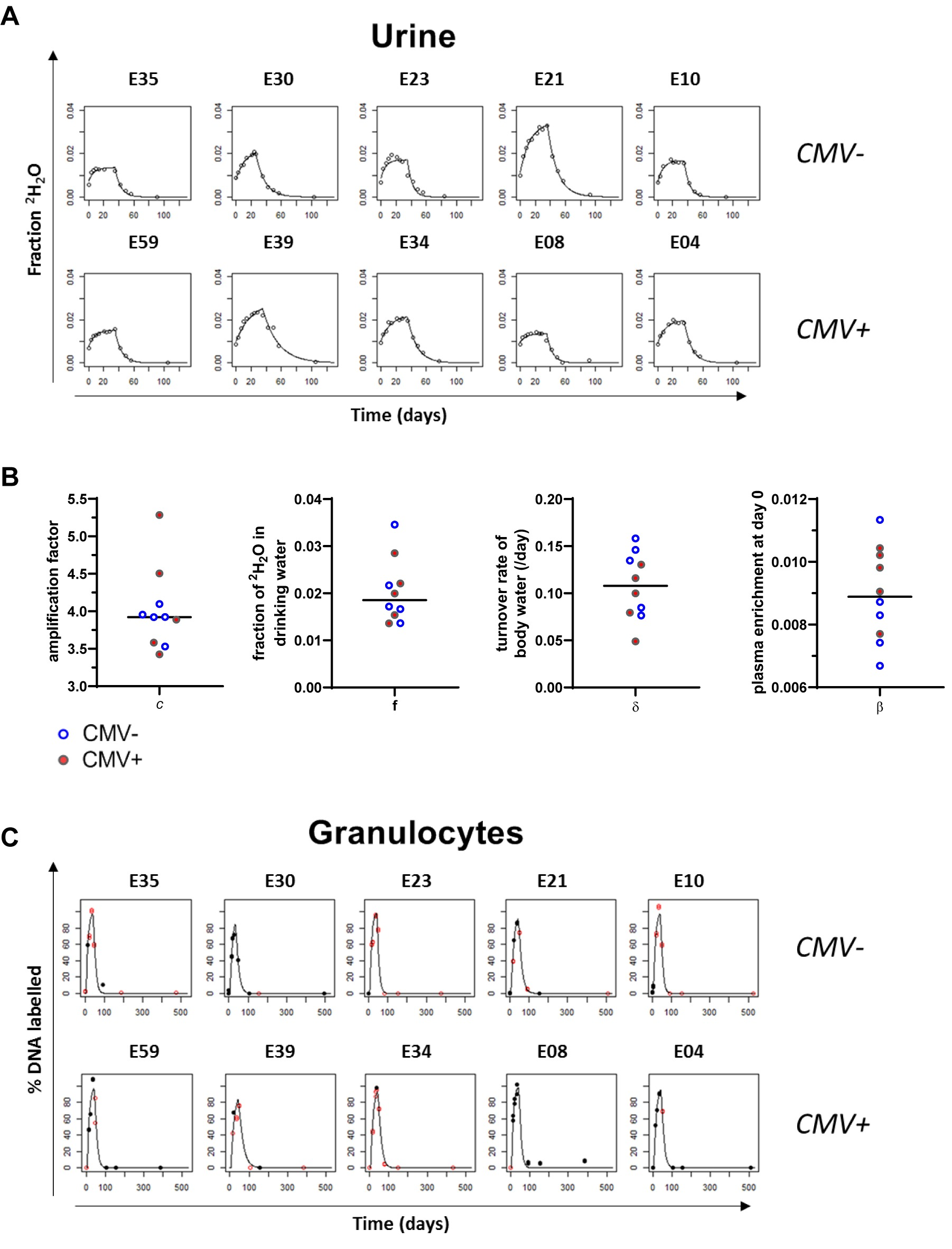

Supplement: S9 Fig — (A) Best fits of the mathematical model to the 2H enrichment in urine (see Materials and methods for differential equations). (B) Parameter estimates of the urine enrichment curves in all participants, where c represents the amplification factor, f the fraction of 2H2O in the drinking water, δ the turnover rate of body water per day, and β the plasma enrichment due to the boost at day 0. (C) Best fits of enrichment curves in granulocytes (see Materials and methods). The measurements that were out of range of the standards are indicated in red closed symbols, those in range are indicated in black closed symbols. E35, E30, E23, E21, and E10 are CMV- individuals, E59, E39, E34, E08, and E04 are CMV+ individuals. E30 only received 2H2O for 3.5 weeks. (TIF) [file ppat.1010152.s009.tif]
