# Supplementary material for: Quantification of T-cell dynamics during latent cytomegalovirus infection in humans
Source: PLoS Pathog. 2021 Dec 16;17(12):e1010152. doi: 10.1371/journal.ppat.1010152 (PMC8717968; doi:10.1371/journal.ppat.1010152)
Supplement: S1 Table — The selected individuals for the deuterated water labelling study are shaded in the table and the selected dextramer+ CMV-specific CD8+ T-cell populations are bold and italic. Individuals were tested with dextramers that corresponded with their HLA-type. 0 = no response to dextramer; NA = not applicable. (DOCX) [file ppat.1010152.s010.docx]

**S1 Table. CMV-specific CD8^+^ T-cell dextramer staining.**

The selected individuals for the deuterated water labelling study are shaded in the table and the selected dextramer+ CMV-specific CD8^+^ T-cell populations are bold and italic. Individuals were tested with dextramers that corresponded with their HLA-type. 0 = no response to dextramer; NA = not applicable.

| **Donor** | **Sex** | **Age** | **Log CMV IgG (MIA)** | **A1-**  **VTE** | **A2-**  **NLV** | **A3-**  **KLG** | **A24-**  **QYD** | **B7-**  **RPH** | **B7-**  **TPR** | **B8-**  **ELR** | **Number of dextramer+ populations** |
| --- | --- | --- | --- | --- | --- | --- | --- | --- | --- | --- | --- |
|  |  |  |  | (Frequency of dextramer+ cells out of total CD8^+^ T-cells.) | | | | | | |  |
| **E02** | Male | 65,5 | 2,05 | 1,59 | 1,78 | NA | NA | NA | NA | NA | 2 |
| ***E04*** | *Male* | *68,8* | *2,27* | ***13,83*** | *NA* | *NA* | *NA* | *NA* | *NA* | ***15,25*** | *2* |
| **E07** | Male | 62,1 | 2,44 | 0 | 0,98 | NA | NA | 0 | 3,68 | NA | 2 |
| ***E08*** | *Male* | *66,6* | *2,28* | *0* | ***4,43*** | *NA* | *0* | *NA* | *NA* | *NA* | *1* |
| **E09** | Female | 69,6 | 2,46 | 0 | NA | NA | 0 | NA | NA | NA | 0 |
| **E17** | Female | 69,8 | 1,23 | NA | NA | 0 | 0 | NA | NA | NA | 0 |
| **E20** | Male | 61,3 | 1,9 | NA | 0 | 0,72 | 0 | NA | NA | NA | 1 |
| **E22** | Female | 68,1 | 2,31 | NA | 1,74 | NA | 0 | NA | NA | NA | 1 |
| **E24** | Female | 67,6 | 1,79 | NA | 0 | 0 | 0 | 0,87 | 0,76 | NA | 2 |
| **E26** | Female | 73,3 | 2,15 | 0 | NA | 0 | NA | NA | NA | NA | 0 |
| **E27** | Male | 63,7 | 1,11 | 4,81 | NA | NA | NA | NA | NA | 1,34 | 2 |
| **E28** | Male | 69,2 | 2,26 | NA | NA | 6,87 | NA | NA | NA | NA | 1 |
| **E31** | Male | 77,5 | 2,24 | NA | NA | NA | NA | NA | NA | NA | 0 |
| **E32** | Male | 76,9 | 1,34 | 0 | NA | NA | NA | NA | NA | NA | 0 |
| ***E34*** | *Male* | *67,7* | *2,16* | *NA* | *5,9* | *NA* | *NA* | *NA* | *NA* | *NA* | *1* |
| **E38** | Female | 64,2 | 2,04 | NA | 0,42 | 0 | 0 | NA | NA | NA | 1 |
| ***E39*** | *Male* | *66,6* | *2,09* | *NA* | *NA* | *0* | *NA* | *0,36* | ***11,25*** | *NA* | *2* |
| **E42** | Female | 79,8 | 2,9 | 0,72 | NA | NA | NA | NA | NA | 5,07 | 2 |
| **E43** | Female | 65,3 | 2,02 | NA | 0,54 | NA | 0 | NA | NA | NA | 1 |
| **E44** | Female | 73,2 | 1,57 | 0 | NA | NA | 0 | NA | NA | NA | 0 |
| **E45** | Male | 67,4 | 1,07 | 0 | NA | NA | NA | NA | NA | 0 | 0 |
| **E48** | Female | 68,0 | 2,19 | 3,61 | 6,1 | NA | 0 | NA | NA | 0 | 2 |
| **E49** | Male | 75,7 | 2,31 | NA | 2 | NA | 0 | NA | NA | NA | 1 |
| **E51** | Female | 68,3 | 2,12 | NA | NA | 3,15 | NA | NA | NA | NA | 1 |
| **E53** | Male | 69,3 | 1,98 | NA | 0 | NA | NA | NA | NA | NA | 0 |
| **E54** | Female | 74,9 | 2,06 | NA | 2,39 | NA | 0 | NA | NA | 0 | 1 |
| **E55** | Female | 72,7 | 1,95 | 3,28 | NA | NA | NA | 0 | 0 | NA | 1 |
| **E56** | Male | 69,1 | 1,5 | NA | 0,19 | NA | 0 | NA | NA | NA | 1 |
| **E57** | Male | 67,8 | 2,39 | NA | 0,99 | 0 | NA | NA | NA | NA | 1 |
| **E58** | Male | 63,2 | 2,19 | 1,37 | NA | 0 | NA | NA | NA | 0 | 1 |
| ***E59*** | *Female* | *76,0* | *3,51* | *NA* | *0* | *0* | *0* | *0,81* | ***15,2*** | *NA* | *2* |
| **E60** | Male | 61,0 | 2,05 | NA | NA | NA | 0 | NA | NA | 0 | 0 |
|  |  |  |  | **Total dextramer+ CD8^+^ T-cell populations** | | | | | | | 32 |
|  |  |  |  | **Total number of CMV+ participants** | | | | | | | 32 |
