# Supplementary material for: Quantification of T-cell dynamics during latent cytomegalovirus infection in humans
Source: PLoS Pathog. 2021 Dec 16;17(12):e1010152. doi: 10.1371/journal.ppat.1010152 (PMC8717968; doi:10.1371/journal.ppat.1010152)
Supplement: S2 Table — (DOCX) [file ppat.1010152.s011.docx]

**S2 Table. Median (range) of loss rates (*d**) of labelled cells of different T-cell subsets.**

|  | **Median (range) of loss rates (*d**) per day** | | |
| --- | --- | --- | --- |
|  | *All individuals* | *CMV- individuals* | *CMV+ individuals* |
| **CD4^+^ T_CM_ cells** | 0.01534 (0.00701 – 0.02655) | 0.01554 (0.01310 – 0.01921) | 0.01515  (0.00701 – 0.02655) |
| **CD8^+^ T_CM_ cells** | 0.00617 (0.00200 – 0.15910) | 0.00442 (0.00200 – 0.15910) | 0.00639 (0.00452 – 0.02026) |
| **CD4^+^ T_EM/EMRA_ cells** | 0.01240 (0.00305 – 0.03163) | 0.01676 (0.01007 – 0.03163) | 0.00937 (0.00305 – 0.01457) |
| **CD8^+^ T_EM/EMRA_ cells** | 0.00305 (0.00160 – 0.01517) | 0.00250 (0.00195 – 0.01517) | 0.00494 (0.00160 – 0.00707) |
| **CMV-specific CD8^+^ T-cells** | 0.00504 (0.00200 – 0.01860) | N/A | 0.00504 (0.00200 – 0.01860) |
