# Supplementary material for: Quantification of T-cell dynamics during latent cytomegalovirus infection in humans
Source: PLoS Pathog. 2021 Dec 16;17(12):e1010152. doi: 10.1371/journal.ppat.1010152 (PMC8717968; doi:10.1371/journal.ppat.1010152)
Supplement: Attachment — Submitted filename: Response to reviewers.pdf [file ppat.1010152.s013.pdf]

We would like to thank the reviewers for their constructive feedback. We have amended the manuscript according to the suggestions that were made. Please find our point-by-point response to the reviewers' questions below.

Response to Reviewers' Questions

Reviewer #2: The authors have addressed all of my concerns except the first major concern, on the tSNE analysis in Fig. 1E-1G. The conversation was:

Q. The authors show in Figures 1E and 1G MFI-based t-SNE cluster analyses that show distinct clustering of CD8 T cells recognizing CMV-specific immunodominant epitopes. This is highly interesting and relevant, but the paper would benefit from a more detailed representation of the markers showing differences between CMV-specific cells and general TEMRA or EM cells in two-dimensional representative dotplots.

A. We have analyzed the differences between CMV-specific T-cells and bulk TEMRA, TEM and TCM cells using conventional gating in two-dimensional plots for the highly relevant markers CD28, CD57, and KLRG-1 (Figure 2D), which are often associated with CMV-seropositivity and indicative of their phenotype. We used multidimensional t-SNE analysis to investigate specifically whether the differences between the T-cell pools of CMV- and CMV+ subjects could be explained by the sheer presence of large, immunodominant CMV-specific T-cell populations in CMV+ individuals. We feel that following up the multi-dimensional t-SNEanalysis by conventional flow cytometric gating in two dimensions, would in a way be a step backward, and that providing individual dot plots would have limited added value as this would rely on a few representative dot-plots.

I concede that the dot-plots may be a step back, and that the heatmaps provided in figure 1F and 1H provide a more detailed information on the make-up of the tSNE subsets than the representative dot-plots could. Nevertheless, the discussion reveals the problem that well-intentioned readers will have to comprehend the data provided by the non-intuitive tSNE plots. In light of that, it would help the readers (and the authors) if the paper would provide in a separate panel the information on the percentage of cells, within the tetramer labelled groups, that belong to each of the the nine tSNE clusters in the individual cell donors. This information will help us to assess if the information from the representative tSNE in 1G is reproduced in the majority of the individuals, or if variations are more common. This is particularly important because the cluster 9 features a phenotype that is not typically associated with TEMRA cells in inflationary CD8 responses to CMV infection, yet seems to be the dominant one among the antigen specific cells in the representative tSNE in 1G.

Author's response:
We thank the reviewer for clarifying the comment concerning the t-SNE analysis. We agree that it is important to address the representativeness of the different clusters. We have added the distribution of CMV-specific $CD8^+$ T-cells over all nine clusters to Supplementary Figure 2 (in which the distribution of bulk $CD8^+$ T-cells for CMV- and CMV+ individuals was already shown). This additional figure shows that cluster 9 is not unique to a few individuals, but a conserved and dominant phenotype of CMV-specific T-cells in the majority of CMV+ individuals. We agree that the phenotype is not typical for $CD8^+$ T_{EMRA} cells, and have added this notion to the main text.

Reviewer #1: on page 20 the authors mentioned a manuscript as submitted, please check here if PLos allows this statement. If the authors what to hold the statement in the body of the text, I would recommend to publish the submitted manuscript as a Preprint and include it in the reference list.

Author's response:

In the revised version of the manuscript, we refer to the preprint of this work, which has been published in BioRXiv.
