# Supplementary material for: Quantification of T-cell dynamics during latent cytomegalovirus infection in humans
Source: PLoS Pathog. 2021 Dec 16;17(12):e1010152. doi: 10.1371/journal.ppat.1010152 (PMC8717968; doi:10.1371/journal.ppat.1010152)
Supplement: Attachment — Submitted filename: Response to reviewers.docx [file ppat.1010152.s012.docx]

**Response to reviewers**

**Part I - Summary**

Please use this section to discuss strengths/weaknesses of study, novelty/significance, general execution and scholarship.

**Reviewer #1:** The manuscript by van den Berg et al. investigates the dynamic of T cells in older people latently infected with human cytomegalovirus (HCMV). The authors describe marker distribution on HCMV-specific and bulk T-cells 45 persons and analyzed the dynamics of T cell memory subsets for up to 500 days by in vivo deuterium labeling in a smaller proportion. Here, the authors showed that the dynamics of CMV-specific and bulk memory T cells were comparable in both populations and CMV-specific T cells have no longer life-span than the bulk memory T cell population.

The manuscript is well written and the authors discussed in detail their findings and the limitation of the study. The presented experimental data support the drawn conclusions, but remain mostly descriptive. Due to the high interest for immunologists and virologists in the phenomenons of memory inflation of CMV-specific cells and the potential role of CMV in immunosenescence the manuscript should be considered for acceptance in PLoS pathogens after targeting the following points.

**Reviewer #2:** I read with interest the paper “Quantification of T-cell dynamics during latent cytomegalovirus infection in humans” by van den Berg et al. The authors study T-cell dynamics in healthy CMV seropositive people and show that the CD4 compartment is affected by CMV latency more vigorously than the CD8 subset, that immunodominant CMV-specific CD8 cells possess an overall distinct phenotype than the general TEMRA or EM counterparts and that the populations of CMV specific cells are rather stable in the long run. The paper is generally well written, and the results well interpreted. The discussion is somewhat long, but it covers many aspects that need to be addressed to qualify the results and put them in context of the literature and of the scientific consensus on CMV immunity and aging.

**Reviewer #3:** This is an interesting study that addresses a range of features regarding CD4+ and CD8+ T cells in relation to CMV serostatus.

There are interesting results within the work but it will be important to bring out the novelty and limitations of the work.

A concern is that the clarity of the writing is somewhat vague and the scientific message is not clear. The title and abstract are somewhat reflective of this.

**Author’s response:**

We would like to thank the three reviewers for their constructive feedback, which we feel has led to significant improvement of the manuscript. As indicated by Reviewer 1 our conclusions remain largely descriptive. We feel this is somewhat inherent to studies in humans, but agree that these insights from humans are important for researchers interested in memory inflation and CMV infection. We agree with Reviewer 2 that the Discussion is quite lengthy, but also that this is needed to reliably interpret our data and put them into the literature context. In response to Reviewer 3, we have critically looked where we could improve the clarity of the scientific message and highlight more clearly the novelty and limitations of this work. We very much appreciate the suggestion of this reviewer to summarize our findings in a cartoon, which we have included as Figure 6 in the revised version of the manuscript.

**Part II – Major Issues: Key Experiments Required for Acceptance**

Please use this section to detail the key new experiments or modifications of existing experiments that should be absolutely required to validate study conclusions.

Generally, there should be no more than 3 such required experiments or major modifications for a "Major Revision" recommendation. If more than 3 experiments are necessary to validate the study conclusions, then you are encouraged to recommend "Reject".

**Reviewer #1:** **Major issues**

1. As far as I understand the authors the authors only used selected multimers specific for a defined combination of peptide and HLA-molecule to analyze the CMV-specific response in the donors. The multimers can detect epitopes that are immunodominant from the total population point of view. Nevertheless, it is known from the literature that the individual response against CMV is highly variable (for analyses see Sylwester et al. (already cited) Toya et al. PMID: 33335252, Attaf et al. PMID: 3310126, Gabel et al PMID: 3310126, Smith et al. PMID: 3310126) and therefore the author will miss most of the CMV-specific response in their analyses. They mentioned this problem in the discussion, but do not target it in their experiments. I was wondering why they do not try to get a better understanding of the total CMV response by using peptide cocktails or APCs loaded with cell extracts of infected cells to estimate the total response for each donor.

We fully agree that our experiments do not cover the full T-cell response to CMV, and that responses to subdominant epitopes may be missed in our analyses. In fact, we deliberately focused on immunodominant immune responses for two reasons: 1. A key research question was whether the observed differences in the total T-cell pool between CMV+ and CMV- individuals could be a direct consequence of the presence of large, immunodominant CMV-specific T-cell populations, which could possibly have different characteristics related to their large size. 2. We aimed to investigate how these large CMV-specific T-cell populations maintain themselves. Focusing on immunodominant CMV-specific T-cell responses helped us to address both questions. In the revised version of the manuscript, we now more carefully write that our findings pertain to the impact and characteristics of dominant CMV-specific populations only, and address the discussion around this point in more detail (see also our response to major point 2 of Reviewer 2).

Importantly, the typical characteristics of the large CMV-specific T-cell populations that we investigated appeared to be very consistent between individuals and for 6 different HLA-peptide specificities. We have addressed this in more detail by adjusting Fig 1G, which now shows both the fraction of CMV-specific T cells per individual in cluster 9 and clusters 5-8. This shows that the observed patterns in the t-SNE plots of Fig 1G are not caused by a few exceptional responses, but are truly characteristic of the immunodominant CMV-specific T-cell responses. Taken together, these findings strengthen our conclusion that the observed differences between CMV+ and CMV- individuals as observed in the t-SNE plots of Fig 1E are not explained by the sheer presence of large numbers of immunodominant CMV-specific T cells.

2. The authors selected for the in vivo labeling the 5 donors with the highest number of multimer-positive CD8 T cells, because they hypothesize that any difference would be more obvious in these donors. But it also could be that in these the differences might be less pronounced because here a steady-state situation has been already reached. When the authors are only interested in the maintenance of the memory pool it is fine to look at this situation. But when they also would be interested in the expansion of the memory pool (memory inflation), it would be better to include also donors with lower frequencies of epitope-specific T cells, because here the expansion of a limited number of T-cells clone might be better to observe.

Our focus on the largest CMV-specific T-cell responses in elderly people may indeed have led to a focus on inflated T-cell responses that have already reached their maximum size. In line with this, over the time frame of the experiment, we did not observe any significant increases in CMV-specific T-cell numbers in these individuals. We agree with the reviewer that our results thereby provide information about the maintenance, and not the development, of large CMV-specific T-cell responses. We describe this more precisely in the revised version of the manuscript.

**Reviewer #2: Major issues**

Overall, the paper is highly interesting for experts studying CMV immunity and of interest for the more general audience of immunological gerontologists. However, some aspects need to be addressed before the paper may be published in PLOS Pathogens.

1. The authors show in Figures 1E and 1G MFI-based t-SNE cluster analyses that show distinct clustering of CD8 T cells recognizing CMV-specific immunodominant epitopes. This is highly interesting and relevant, but the paper would benefit from a more detailed representation of the markers showing differences between CMV-specific cells and general TEMRA or EM cells in two-dimensional representative dotplots.

We have analyzed the differences between CMV-specific T-cells and bulk T_EMRA_, T_EM_ and T_CM_ cells using conventional gating in two-dimensional plots for the highly relevant markers CD28, CD57, and KLRG-1 (Figure 2D), which are often associated with CMV-seropositivity and indicative of their phenotype. We used multidimensional t-SNE analysis to investigate specifically whether the differences between the T-cell pools of CMV- and CMV+ subjects could be explained by the sheer presence of large, immunodominant CMV-specific T-cell populations in CMV+ individuals. We feel that following up the multi-dimensional t-SNE-analysis by conventional flow cytometric gating in two dimensions, would in a way be a step backward, and that providing individual dot plots would have limited added value as this would rely on a few representative dot-plots.

2. The authors conclude that “the changes in the phenotype of the T-cell pool of CMV+ individuals cannot solely be explained by the presence of large numbers of CMV-specific T-cells.” They concede in the discussion that it cannot be excluded that CD8 recognizing subdominant CMV antigenic epitopes may have a phenotype that is closer to the general CD8 population, but dismiss the scenario, arguing that they used tetramers against 7 most abundant epitopes. This is an over interpretation, because they did not test if other HLA molecules that were present in their test subjects may carry additional antigenic epitopes. Furthermore, it is known that subdominant epitopes have phenotypes that are distinguishable from the dominant ones and in light of the huge genomic size of CMV it is impossible to exclude that numerous additional epitopes contributed to the TEMRA pool and formed it. Therefore, this argument needs to be altered in the final paragraph of the introduction and in the abstract, introducing the alternative scenario as delineated above.

We agree with the reviewer that we have been unclear here, and have adapted the text to make the conclusions clearer and to avoid over-interpretation. Our line of argument, which we follow more clearly in the revised manuscript, is as follows: Our observation (in line with previous observations by others) is that the T-cell pool of CMV+ individuals is clearly distinct from that of CMV- individuals. Although it is tempting to speculate that these differences are due to the effect of CMV-infection on the whole T-cell pool (as has been suggested by e.g. Weltevrede et al. Exp Gerontol., 2016), we addressed whether they could alternatively be the direct result of the presence of large, inflated CMV-specific T-cell responses, which themselves may have characteristics different from T-cells with other specificities. One of our major results is that the differences in the T-cell pool between CMV+ and CMV- individuals cannot be fully explained by the presence of large, immunodominant CMV-specific T-cell responses, thereby rejecting our null hypothesis. This leaves open two possibilities: The first is that CMV infection indeed affects a lot of T-cells in the pool, even those that are not CMV-specific, as we have shown previously for EBV-specific T-cells (Lanfermeijer et al. Front. Immunol., 2021). The second possibility is that small CMV-specific T-cell clones have characteristics that differ from those of large, inflated CMV-specific T-cells and from those of T-cells with other specificities, and that those smaller CMV-specific T-cell populations explain the differences observed between CMV+ and CMV- individuals. Although we consider the latter option less likely, our analyses cannot distinguish between these two possibilities. We discuss this more precisely and clearly in the revised version of the manuscript, and removed the statement from the Discussion that our findings show that CMV influences T-cells with other specificities, which was indeed an over-interpretation.

3. In figure 3C, the authors show several comparisons and one that produced a p=0.095 in the CD4 TEM/EMRA fraction. It is not clear if they corrected the stat analysis for the number of tests performed, which would raise the p value and void this claim. If a Bonferroni-like correction was performed, the argument may stand, but if not, the authors are advised to avoid this claim. In any case, given the rather high level of the p values, the wording may benefit from a more neutral assessment (e.g.: Mathematical modelling supported a trend towards a lower production)

The apparent difference between CD4 T_EM/EMRA_ cells between CMV- and CMV+ individuals was highlighted for several reasons: 1. Even though the calculated in vivo production rates did not reach statistical significance within this limited number of participants, the shape of the deuterium labelling curves is strikingly different, showing steep up- and downlabelling for CMV- but not CMV+ individuals. 2. In the larger group of individuals in figures 1 and 2, the difference in senescence and lifespan-associated markers pointed in the same direction of increased lifespans of CD4 T_EM/EMRA_ cells in CMV+ versus CMV- individuals. However, the reviewer is right in that the statistical analysis of figure 3C was not yet corrected for multiple testing. We have added this in the revised manuscript using a non-parametric Kruskal-Wallis test. This yielded a non-significant *p*-value. Therefore, we have now phrased the observation in a more neutral manner throughout the manuscript.

**Reviewer #3: Major issues**

Much is made of the statement: "The impact of CMV infection on the T-cell pool

stretches beyond the presence of large CMV-specific CD8+ T-cell populations". What does this mean ? Does it suggest that T cells specific for other antigens also have altered phenotype in CMV+ people? As the authors state, there will be large populations of CD8+ cells that are specific for CMV but are not detected by the dextramers. This could easily be the explanation. Did the authors consider the use of dextramers against other viral epitopes to test this theory? The statement appears overstated.

We agree with Reviewer 3 that the cited statement was somewhat ambiguous. We would kindly like to refer to our answers to Reviewer 1 (Major issue 1) and Reviewer 2 (Major issue 2), as the raised points are largely overlapping. Briefly, in the manuscript we show that the differences in the phenotype of the T-cell pool between CMV+ and CMV- individuals cannot be explained by the presence of large immunodominant CMV-specific T-cell populations. The remaining differences could be a consequence of both explanations raised by Reviewer 3. No other epitopes were tested as this was not the scope of the paper, but other work by us (Lanfermeijer et al. Front. Immunol., 2021) and others (Khan et al. PMID: 12165524) has shown an influence of CMV infection on heterologous T-cells. We have clarified the statement throughout the manuscript and altered the discussion to include these explanations more clearly.

The novel aspects of the work need to be addressed. There are very many papers on immunophenotyping of CMV-specific T cells and the tSNE plots do not add substantially. What are the major novel points?

Our paper is not just about immunophenotyping of CMV-specific T-cells and stretches beyond the analyses that are usually performed. Our novel way of visualizing the CMV-specific T-cells amongst the total T-cell pool using t-SNE analysis clearly shows the complex manner in which CMV is contributing to changes to the total T-cell pool. Our data indicate that the highly researched immunodominant CMV-specific T-cells are not the reason for the general changes in the T-cell pool established by CMV infection.

In addition, we studied a novel aspect of the CMV-specific T-cell population as well as the memory compartments in CMV- versus CMV+ individuals by quantifying the *in vivo* lifespan of these T-cells in humans. Two previous studies showed that antigen-specific T-cells are long-lived in the memory phase (Akondy et al. PMID: 29236685; Wallace et al. PMID: 20738423), and that CMV-specific T-cells may even greatly outlive non-CMV-specific T-cells (Wallace et al. PMID: 20738423). We now show that CMV-specific T-cells in fact have comparable lifespans to bulk memory T-cells.

The reduction of Ki-67 on CD4+ cells in CMV+ people seems novel and perhaps is a major point of the paper. Is there a hypothesis for this?

The impact of CMV infection on the expression of senescence markers and the reduction in Ki-67 on CD4^+^ T-cells was indeed surprisingly large. The trend toward lower deuterium incorporation of CD4^+^ T_EM/EMRA_ cells in CMV+ compared to CMV- individuals seems to point in the same direction of a longer lifespan of CD4+ T_EM/EMRA_ cells in CMV+ individuals. Generally, senescence-associated markers are expressed at much higher levels by CD8+ as compared to CD4^+^ T-cells, both in CMV- and CMV+ individuals (see Figure 1B). We also know that CD8^+^ T-cells live longer than CD4^+^ T-cells (Figure 3C, but also described in a review by Macallan et al. PMID 31922055 summarizing results from multiple labs). We hypothesize that cytotoxic potential itself is related to longevity, which would be in line with the presence of cytotoxic CD4^+^ T-cells in CMV infection. We have added this hypothesis to the discussion.

I have concerns that the analysis is somewhat overstated. The p-value for reduction in average production/loss rate of CD4+ TEM/EMRA cells in CMV+CD4 proliferation is 0.1 but this association has a results paragraph and is commented on many times as 'trend', 'hint'. A one sentence note of the trend is all that is needed and the rest should be excluded.

We understand the concerns of the reviewer, which were also shared by Reviewer 2 (Major issue 3). In this manuscript, we show that the phenotype of CD4^+^ T_EM/EMRA_ cells between CMV- and CMV+ individuals are strikingly different. In CMV+ individuals, a significantly higher percentage of CD4^+^ T-cells express the senescence markers CD57 and KLRG-1 whereas CD28 is downregulated (Figure 1B). Combining these lifespan-associated markers with the difference in Ki-67 expression (Figure 2A), our principal component analysis points towards an increased lifespan of CD4^+^ T_EM/EMRA_ cells in CMV+ compared to CMV- individuals (Figure 2B). The slow up- and downlabelling of CD4^+^ T_EM/EMRA_ cells in CMV+ individuals compared to CMV- individuals, indicative of a longer lifespan, is in agreement with these other observations. However, statistical analysis corrected for multiple testing rendered this difference insignificant. Thus, we have put less emphasis on this statement in agreement with the reviewer’s comment.

In addition, the positive association of deuterium proliferation with Ki67 is only made by exclusion of one subject, which appears inappropriate.

Both with and without this subject (E04), we observed a significant correlation between expression of the proliferation marker Ki-67 and *in vivo* lifespans as estimated by deuterium labelling, and for both situations we reported *p*-values and correlation coefficients. E04 puts forward an interesting example highlighting the difference between both methods to assess proliferation. Stable isotopes like deuterium can be incorporated throughout the body and this method is not limited by the location at which cells divide. A static marker like Ki-67, however, reflects the proliferation state at a single moment and location. Hence, it is more sensitive to day-to-day variations and intermittent immune activations (as we describe in the discussion). We therefore would like to keep this example in, though we have now phrased the results in a more neutral way.

**Part III – Minor Issues: Editorial and Data Presentation Modifications**

Please use this section for editorial suggestions as well as relatively minor modifications of existing data that would enhance clarity.

**Reviewer #1: Minor points**

1. The rationale of analyzing memory dynamics only in older persons remains unclear.

We chose to study the memory dynamics in older persons for two reasons. Firstly, we hypothesized that the effects of CMV would be the largest in older individuals who have the highest chance of having had CMV infection for a long time. In addition, the effect of CMV on CD8^+^ T_EM_ expansion was described to be the largest for older individuals (Wertheimer et al. PMID: 24501199). Secondly, in our previous study (Westera et al. PMID: 25627171) we observed a much higher T-cell proliferation rate in the one older adult who was CMV+. We have made these reasons clearer in the revised manuscript.

2. The authors mentioned in the third paragraph of the discussion the role of reactivation for the establishment of the unique CMV-specific memory. The term reactivation implies for the broader audience production of viral progeny, but in the last years, it comes more clear that transcriptional activity of latently infected cells might be the reason for T cell expansion during viral latency. Please see work by the labs of Felicia Goodrum, Mark Wills, John Sinclair (all for HCMV), Matthias Reddehase (for MCMV).

We totally agree with the reviewer and have now referred to it as ‘partial' reactivation in the discussion.

3. In the same paragraph, the authors define “Memory Inflation” as an increase of memory T cells. Specifically for mice, it is clear that in animals with a high latent load (after high dose infection or after HCT) the inflationary cells are effector cells with some memory cell-like features (Baumann et al. PMID: 29652930), true Inflation of memory cells was only recently shown by Holtappels et al. PMID: 32707744.

We define memory inflation as an increase in “memory” T-cell numbers over time, where “memory” refers to all types of antigen-triggered, non-naive cells, including the effector/T_EMRA_ cells which are so typical for CMV infection. Indeed, we also observed these different phenotypes within the CMV-specific T-cell population (Figure 1C). We thank the reviewer for the recent paper by Holtappels et al. and have added this reference to the introduction as well as a clearer definition of “memory”.

4. In figure 4A short time increase and decrease of total T cells and frequency are visible in some of the donors. Do the authors have any explanation, why this is not reflected in the subfigure 4B.

The short-time increases and decreases in CMV-specific T-cell numbers are directly related to fluctuations in the total number of T-cells at the time of blood withdrawal. Notably, the *percentages* of CMV-specific T-cells in the total CD8^+^ T-cell pool were much more stable. For Supplementary Figure 4B (and also Figure 1A), we chose to display a median for individuals for whom repeated measurements were available (i.e. the individuals participating in the heavy water labelling study), to even out such fluctuations.

5. In the discussion; I miss at some point the inclusion of the knowledge obtained from MCMV infected mice (4th paragraph, last two paragraphs).

Although we fully agree that knowledge obtained from MCMV-infection in mice is of interest, we deliberately chose to focus this manuscript on CMV infection in humans as we have now emphasized more in the 3^rd^ and 4^th^ paragraph of the introduction. We feel that the manuscript is already quite lengthy (see also the General comment of Reviewer 2). We have referred to the insights obtained from mice where they directly link to our work (paragraph 5), but have otherwise referred to reviews on MCMV.

**Further comments**

1. In the paragraph PBMC, neutrophilic granulocytes, and serum isolation and storage, please use molarity/concentration and not weight for the buffer composition

2. In some of the figures S5 & S6, it is hard to assign the donors to the CMV+ or CMV- group. Please make it more clear.

**Reviewer #2:** **Minor points**

In general, the authors should adapt statements like “Based on t-SNE analysis, we found that CMV-specific CD8+ T-cells only partially explain the differences …” into something like “Based on t-SNE analysis, we found that IMMUNODOMINANT CMV-specific CD8+ T-cells only partially explain the differences …”

We agree with the comments above from Reviewers 1 and 2 and have changed the manuscript at these points.

**Reviewer #3: Minor points**

It might be helpful for the authors to consider what the take home messages are from the paper and the messages that would get this cited. That could help the writing style. Would a summary cartoon be useful?

We have written a clearer paragraph on the aim and take-home messages of the paper and have followed the very valuable suggestion of this reviewer to add a cartoon (Figure 6 of the revised manuscript), summarizing the main messages of the paper.

One final correction we made in the revised manuscript addresses the best fits of individual E08. In the original manuscript, these fits did not always look very convincing. We found out that this was due to a technical issue, related to the lack of data points at time 0 for this individual. We have corrected the fits, estimates, and statistics in the new version of the manuscript (Fig. 3C, Fig. 4C/D, Fig. 5, Table 2, Sup. Fig. 6/7/9).

PLOS authors have the option to publish the peer review history of their article. If published, this will include your full peer review and any attached files.

If you choose “no”, your identity will remain anonymous but your review may still be made public.

**Do you want your identity to be public for this peer review?** For information about this choice, including consent withdrawal, please see our [Privacy Policy](https://www.plos.org/privacy-policy).

Reviewer #1: No

Reviewer #2: **Yes:** Luka Cicin-Sain

Reviewer #3: No

Figure Files:

While revising your submission, please upload your figure files to the Preflight Analysis and Conversion Engine (PACE) digital diagnostic tool, [https://pacev2.apexcovantage.com](https://pacev2.apexcovantage.com/). PACE helps ensure that figures meet PLOS requirements. To use PACE, you must first register as a user. Then, login and navigate to the UPLOAD tab, where you will find detailed instructions on how to use the tool. If you encounter any issues or have any questions when using PACE, please email us at figures@plos.org.

Data Requirements:

Please note that, as a condition of publication, PLOS' data policy requires that you make available all data used to draw the conclusions outlined in your manuscript. Data must be deposited in an appropriate repository, included within the body of the manuscript, or uploaded as supporting information. This includes all numerical values that were used to generate graphs, histograms etc.. For an example see here on PLOS Biology: <http://www.plosbiology.org/article/info%3Adoi%2F10.1371%2Fjournal.pbio.1001908#s5>.

Reproducibility:

To enhance the reproducibility of your results, we recommend that you deposit your laboratory protocols in [protocols.io](http://protocols.io/), where a protocol can be assigned its own identifier (DOI) such that it can be cited independently in the future. Additionally, PLOS ONE offers an option to publish peer-reviewed clinical study protocols. Read more information on sharing protocols at <https://plos.org/protocols?utm_medium=editorial-email&utm_source=authorletters&utm_campaign=protocols>
